# AQbD-enhanced green RP-UPLC-PDA methodology for quantification and forced degradation studies for omeprazole, amoxicillin, and rifabutin

**DOI:** 10.1186/s13065-024-01337-6

**Published:** 2024-11-18

**Authors:** S. P. Ashnah Baffinsha, Vijayageetha Ragupathi, Hemanth Kumar Chanduluru

**Affiliations:** 1 Department of Pharmaceutical Analysis, C.L Baid Metha College of Pharmacy, Thoraipakkam, Chennai, 6000097 India; 2 Department of Pharmaceutical Research, SRM College of Pharmacy, SRM Institute of Science and Technology, Kattankulathur, Chennai, 603203 India

**Keywords:** RP-UPLC, Analytical quality by design, Omeprazole, Amoxicillin, Rifabutin, Green analytical chemistry

## Abstract

The ternary combination like omeprazole (OMP), amoxicillin (AMX), and rifabutin (RFB) was approved by the FDA in November 2019 for combating Helicobacter pylori infections and ulcers caused by this infection. This study aims to develop and authenticate a robust and eco-friendly RP-UPLC technique aimed at the concurrent analysis of OMP, AMX, and RFB, following ICH guidelines, Analytical Quality by Design (AQbD), and green analytical chemistry (GAC) principles. The analysis used the Thermo C18 column (100 mm × 2.1 mm, 1.7 µm), ethanol, and formic acid solution (43:57) as mobile phase with a flow rate of 0.2 ml/min at 272 nm. The method was developed based on the ICH Q14 and validated according to ICH Q2(R1) followed by Forced degradation studies under various conditions. The method showed good linearity for OMP, AMX, and RFB, with coefficient of determination (r2) of 0.9995, 0.9993, and 0.9997, respectively. Precision studies indicated low %RSD values, confirming high reproducibility. Forced degradation studies confirmed the stability of the drugs for 30 min in acid, base, and redox reactions, and they were also stable for 6 h at 105 °C in dry conditions. GAPI assessment depicted a green and yellow pictogram, AGREE scored 0.85, BAGI scored 80, and RGB12 Whiteness Assessment Tool scored 97.5%. The developed RP-UPLC-PDA technique is robust and reliable for the concurrent quantification of the triple combination. It aligns with sustainability goals, enhancing the efficiency and environmental sustainability of pharmaceutical analysis, and setting a benchmark for future analytical methods.

## Introduction

Infection with Helicobacter pylori, commonly referred to as H. pylori, is highly prevalent worldwide. This bacterium is a major cause of stomach and small intestinal ulcers, infecting approximately 40–50% of the global population. H. pylori as a Group 1 carcinogen has been identified by the World Health Organization (WHO) owing to its strong connection with stomach cancer development. Experimental research has shown that eliminating H. pylori can significantly lower the incidence of stomach malignancy in individuals, highlighting its preventative potential [1–3]. A global panel of experts has recommended specific protocols for the eradication of H. pylori, with the updated Maastricht guidelines supporting previous treatment recommendations. The primary components of the standard triple therapy include omeprazole (OMP), amoxicillin (AMX), and rifabutin (RFB) [4–6]. This regimen involves taking four capsules every 8 h with food for a duration of fourteen days. In November 2019, the FDA approved Talicia capsules as a treatment for Helicobacter pylori infections. These delayed-release tablets provide a fixed triple dose of OMP (10 mg), RFB (12.5 mg), and AMX (250 mg). The approval of Talicia pills represents a significant advancement in the management of H. pylori infections. Effective treatment regimens, like Talicia, offer a promising solution for those affected by this pervasive pathogen. [7, 8]

**Fig. 1:**
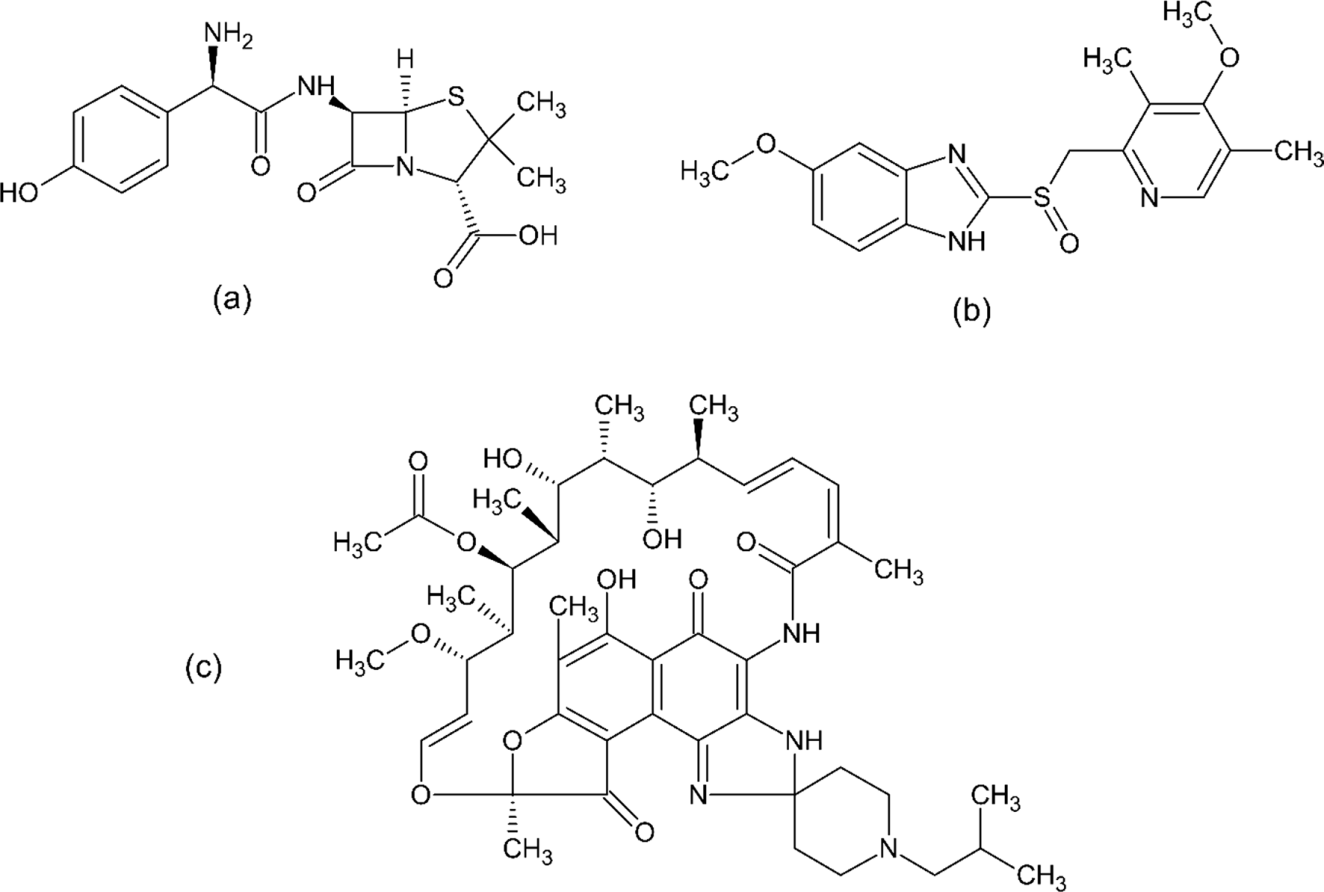
Structure (**a**) AMX, (**b**) OMP, and (**c**) RFB

AMX, a semisynthetic antibiotic from the penicillin class, is acid-stable and used for its bactericidal activity. Its chemical name is (2S, 5R, 6R)-[(2R)-2-amino-2-(4-hydroxyphenyl) acetyl] amino]-6- [[3,3-dimethyl-7-oxo-4-thia-1-azabicyclo [3.2.0] heptane-2-carboxylic acid (Fig. 1a). AMX has a pKa of 2.6 and a log P value of 0.87. It exerts its bactericidal effect by binding to one or more penicillin-binding proteins, thereby inhibiting bacterial cell wall synthesis [9].

Proton pump inhibitors (PPIs) such as OMP are widely used in the treatment of peptic ulcer disease and dyspepsia. Chemically, OMP is known as 6-methoxy-2-[(4-methoxy-3,5-dimethylpyridin-2-yl) methyl sulfinyl]-1H-benzimidazole (Fig. 1b). It has pKa values of 4.77 for its base and 9.29 for its acid, with a log P value of 2.23. Through their interactions with adenosine, PPIs also influence the absorption and metabolism of various medications [10].

RFB, a spiro-piperidyl-rifamycin derivative of rifamycin-S, shares several structural similarities with rifampin and exhibits broad-spectrum antibacterial activity. Chemically, RFB is identified as [9S, 12E, 14S, 15R, 16S, 17R, 18R, 19R, 20S, 21S, 22E, 24Z]-tetrahydroxy-1-isobutyl-14-methoxy-7, 9, 15, 17, 19, 21, 25-tetrahydroxy-6, 16, 18, 20 epoxy pentadeca{1,11,13} trienimino-hepta-methy-spiro{9, 4–2, 3, 7, 8}-naphth{1, 2-d}imidazole-2, 4-piperidin-2H-furo-5,10, 26-(3H, 9H) -trione- 16-acetate (Fig. 1c). Its log P value is 3.2, and it has a pKa of 6.9. RFB works by inhibiting DNA-dependent RNA polymerase in both gram-positive and some gram-negative bacteria, thereby suppressing RNA synthesis and leading to cell death [11]. These pharmacological agents—OMP, AMX, and RFB—form the cornerstone of effective H. pylori eradication therapy, each contributing unique mechanisms to combat the infection and prevent complications such as stomach ulcers and cancer.

Every medication offers unique benefits; however, the treatment of H. pylori infection is most effective with a fixed-dose combination therapy. This combination therapy is administered orally and should only be used for infections confirmed or strongly suspected to be bacterial in nature. Despite its efficacy, this route of administration can lead to some side effects.

Currently, no published analytical method exists for the assay of the OMP, AMX, and RFB combination using the present guidelines, although few methods have been reported either they have used the toxic solvent not adhering with the present updated guidelines [12, 13]. ICH guidelines are recommended to improve the accuracy of UPLC analysis. Additionally, the ICH Q14 guidelines mandate the development of methods built on the philosophies of Analytical Quality by Design (AQbD) [14–16]. This approach aids in creating robust methods and allows for continuous monitoring and improvement under consistent conditions, thereby minimizing the need for revalidation. The aim of this study was to develop an analytical method utilizing AQbD principles to establish a novel UPLC approach. This methodology fosters a collaborative environment for developing effective and environmentally friendly analytical techniques.

## Materials and methods

### Reagents and materials

Biocon Lab generously provided samples of the pure medications OMP, AMX, and RFB, with labeled purities ranging from 97 to 102%. Ethanol (HPLC grade) was sourced from the Hayman Group Ltd. (UK). Additional chemicals used in the study, including sodium hydroxide, formic acid, hydrochloric acid, 30% hydrogen peroxide, potassium dihydrogen orthophosphate (KH₂PO₄), and sodium bisulfate, were procured from SRL (Chennai).

### Instrumentation and chromatographic conditions

The analysis was conducted using the Agilent 1290 Infinity II LC, a UPLC system armed with a Photo Diode Array (PDA) detector. Empower 2 software facilitated both data collection and processing. For chromatographic separation, a Thermo C18 column (100 mm × 2.1 mm, 1.7 µm) was employed. The flow rate was maintained at 0.2 ml/min, with the column temperature kept at ambient conditions. The mobile phase consisted of a formic acid solution and ethanol in a 43: 57 ratio, which provided an optimal reduction in retention (Rt) time and satisfactory resolution (Rs) between AMX, RFB, and OMP [13]. The detection wavelength was optimized at 272 nm. The entire procedure was carried out with a run period of 5 min. All analyses were performed at room temperature.

### Preparation of aqueous phase (formic acid solution)

To prepare the formic acid solution, accurately measure 1.354 ml (obtained from the AQbD Process) of formic acid using a calibrated micropipette with a precision of 0.01 ml. Add the measured formic acid to HPLC-grade water, ensuring that the final volume of the solution is adjusted to 1 L. Thoroughly mix the contents to ensure homogeneity. The solution is then filtered through a 0.22 μm membrane filter using a vacuum filtration system to remove any particulate matter. Finally, sonicate the filtered solution to further ensure it is free of particulates and to enhance clarity.

### Preparation of standard and sample

Precisely measured amounts of 20 mg of OMP, 500 mg of AMX, and 25 mg of RFB were added to a 100 ml volumetric flask. These were dissolved in ethanol and sonicated for 10 min and then diluted with ethanol to reach the 100 ml mark, creating the stock solution. From this stock solution, 1 ml was transferred to a 10 ml volumetric flask and diluted to the final volume with the mobile phase. The final standard solution was filtered through a polyvinylidene fluoride (PVDF) filter with a 0.22 μm pore size before being placed into the vial.

A batch of granules prepared in-house for capsule filling was taken for analysis. A precisely measured sample matching the label claim was placed into a 10 ml volumetric flask, dissolved in ethanol, and sonicated for 10 min to ensure complete dissolution. The solution was then diluted to the 10 ml mark with ethanol, forming the sample stock solution. From this stock solution, 1 ml was further diluted in a 10 ml volumetric flask with ethanol to create the final sample solution. The resulting solution was filtered through a 0.22 μm PVDF filter before being transferred to a vial for analysis.

### AQbD approach in method development

The method was developed using an AQbD framework, beginning with the identification of Critical Analytical Attributes (CAAs) and Critical Method Parameters (CMPs). This identification process utilized risk assessment tools, specifically an Ishikawa diagram, complemented by expert knowledge. The Ishikawa diagram was used to systematically identify potential sources of variability that might impact method performance. It classifies risks such as materials (e.g., variations in reagents or active pharmaceutical ingredients), methods (e.g., procedural inconsistencies), instruments (e.g., calibration issues), environment (e.g., temperature or humidity fluctuations), and human factors (e.g., operator variability). This structured approach aids in pinpointing critical variables that require control to maintain method robustness.

The Knowledge-Based Assessment complements this by utilizing existing scientific data, literature, and regulatory guidelines to evaluate potential risks. It involves assessing the physicochemical properties of the compounds (e.g., pKa, log P), referencing past experiments under similar conditions, and adhering to regulatory frameworks like ICH Q8 and Q9. This ensures that the method is grounded in well-established knowledge, reducing the likelihood of unexpected challenges.

Together, these tools enable a thorough risk assessment, ensuring the management of variability and the development of a robust, reliable method that meets performance standards. To assess the impact of CMPs on CAAs and ensure method robustness for the present method, a series of experiments was conducted using Design of Experiments (DoE) software. Key parameters, including organic phase composition and formic acid concentration in the aqueous phase, were systematically varied within a predefined design space to optimize Rs and overall method performance. For each design run, the UPLC system parameters were adjusted according to the experimental design generated by the DoE software. To maintain system stability, 10-min blank runs were performed between each experimental run. Samples used in the AQbD process were prepared from standard solutions as described in Sect. "Preparation of standard and sample", with 5 µl injected into the UPLC for each experimental run. The resulting data were analyzed using the DoE software to refine the design space and further optimize the method. This iterative process allowed for a comprehensive exploration of the parameter space, ensuring the development of a robust and efficient analytical method.

### Method validation

The developed method’s reliability can be ensured by performing various parameters such as precision, linearity, accuracy, and specificity according to the ICH Q2 guidelines [17]. System suitability was confirmed by calculating key metrics including the number of theoretical plates, tailing factor, Rt, and peak area from six replicate injections of the standard preparation. These checks ensured that the chromatographic system was functioning optimally before proceeding with the method validation.

In the Validation protocol linearity was calculated between the area and the drug concentration was investigated by the AQbD assisted experimental run. The prepared concentration range of 5–30 µg/ml for OMP, 125–750 µg/ml for AMX, and 6.25–37.50 µg/ml for RFB was prepared from the standard solutions and used to plot the calibration curve between concentrations versus area, and the R^2^ value was calculated. The LOD and LOQ were calculated according to the ICH guidelines for analytical method validation.

The LOD is often calculated using the following formulas:

1 $$\text{LOD}=\frac{3 \times \sigma }{s}$$

2 $$\text{LOQ}=\frac{10 \times \sigma }{s}$$

where:

σ\ sigma = standard deviation of the response (noise).

s = slope of the calibration curve.

Intraday and inter-day precision have been assessed through replicating analysis (n = 6) at quality control concentrations. At the same concentration level, the analysis was run three days in a row for inter-day precision. To perform the intraday precision, each combination was analyzed, each at a different time interval throughout the day.

The accuracy was measured using the conventional addition method to determine the accuracy of the method. First, a known quantity of a standard drug was added to the previously examined sample (3 known concentration levels) at 50 (10 µg/ml for OMP, 250 µg/ml for AMX, and 12.5 µg/ml for RFB), 100 (20 µg/ml for OMP, 500 µg/ml for AMX, and 25 µg/ml for RFB), and 150% (30 µg/ml for OMP, 750 µg/ml for AMX, and 37.5 µg/ml for RFB) levels and injected into the UPLC system.

### Forced degradation studies

Forced degradation studies were conducted on standard solutions of OMP, AMX, and RFB to assess their stability under various stress conditions. For each drug, 1 ml of standard solution (prepared as described in Sect. "Preparation of standard and sample") was transferred into separate 10 ml volumetric flasks. To induce degradation, 1 ml of specific reagents was added to each flask: 0.1 N HCl for acid degradation, 0.01 N NaOH for base degradation, 3% H₂O₂ for oxidative degradation, and 10% NaHSO₃ for reductive degradation. These mixtures were then diluted to volume with the diluent. For photolytic degradation, samples were exposed to 72 lx of light for 6 h, while thermal degradation was induced by exposing samples to 105 °C for 6 h. After exposure to photolytic and thermal conditions, these stressed samples were prepared following the same procedure described in Sect. "Preparation of standard and sample". All stressed samples, including those subjected to chemical, photolytic, and thermal degradation, were subsequently injected into the UPLC system for analysis. The results obtained from these stressed samples were compared with those of a control sample to assess the extent of degradation. This comprehensive approach allows for the evaluation of drug stability under various environmental and chemical stresses, providing crucial information for formulation development and shelf-life determination.

## Results and discussion

This study successfully developed and validated a UPLC method for the simultaneous determination of OMP, AMX, and RFB, integrating AQbD principles with GAC approaches. The optimized method demonstrated excellent performance characteristics while minimizing environmental impact. Optimal chromatographic conditions were established, achieving linearity ranges of 5–30 µg/ml, 25–750 µg/ml, and 6.25–37.50 µg/ml for OMP, AMX, and RFB, with R^2^ values exceeding 0.9992. The method exhibited high precision and accuracy, with intra-day and inter-day precision and accuracy within less than 2% RSD. Sensitivity was notable, with LOD and LOQ values for all analytes. Furthermore, the method proved robust, maintaining stability under varied conditions. The following discussion elaborates on these results, exploring the chromatographic method optimization process, applying AQbD principles, detailed method validation outcomes, and the environmental considerations addressed through GAC. This comprehensive analysis demonstrates the method's suitability for pharmaceutical analysis and highlights the benefits of combining AQbD and GAC approaches in analytical method development.

### Method development phase

During the method development phase, several attributes, including column type, buffer, and temperature, were considered and applied randomly to the UPLC system. Ethanol was consistently used as the solvent due to its biodegradability and eco-friendliness, with a Greenness score of 6.6, close to that of water (7.3). After multiple trials, the ideal method which yields good Rs between three peaks and peak shapes was obtained by using a Thermo C18 column (100 × 2.1 mm, 1.7 μm) at room temperature. The selection of a mobile phase was pivotal in optimizing the resolution of AMX, OMP, and RFB during chromatographic analysis. At lower pH, AMX, having a pKa of 2.6, predominantly exists in its ionized form, resulting in reduced hydrophobic interactions with the stationary phase and facilitating its early elution. Conversely, OMP, with pKa values of 4.77 (base) and 9.29 (acid), remains largely non-ionized, which enhances its retention time due to increased hydrophobicity. RFB, characterized by a pKa of 6.9 and a higher log P value of 3.2, exhibits the longest retention among the three compounds, further contributing to effective separation. This strategic aqueous phase selection allowed for distinct differences in retention times, leading to enhanced resolution and reliable quantification of the analytes. The mobile phase comprised ethanol and formic acid solution, resulting in complete Rs between OMP, AMX, and RFB, along with excellent system suitability parameters. A single analysis was performed with a flow rate of 0.2 ml/min and a sample injection volume of 5 μl. Although multiple wavelengths could be monitored using PDA, focusing on a single wavelength simplifies the method, reduces data complexity, and enhances reproducibility without compromising the accuracy or precision of the method since 272 nm was selected for the determination of three drugs which shows optimal sensitivity at this wavelength. Further, the developed method has been applied to the DoE software for further finding the role of different attributes on responses.

### Application of the developed method in the AQbD process

Multivariate analytical design, such as AQbD, plays a pivotal role in maintaining the lifecycle of the analytical procedure development process, ensuring quality, consistency, and regulatory compliance [18].

#### Defining analytical target profile (ATP)

The selection of an ATP is crucial for setting the study’s objectives. In this investigation, the primary ATP was to develop an analytical method characterized by precision, accuracy, and low detection limits. A significant challenge in this combination arose from the market formulation composition, where the concentration ratios varied considerably: AMX at 250 mg, while the other two drugs were present at 10 mg and 12.5 mg. Optimizing a method with good system suitability parameters while adhering to GAC principles presented a formidable challenge. Consequently, the ATP was established to develop a method that complies with both regulatory standards and GAC principles.

#### Risk assessment and identification of CMPs

Risk assessment in this study was conducted using two primary approaches: the Ishikawa fishbone diagram (IFBD) and knowledge-based assessment. These methods were employed to determine the CMPs for this study. Initial trials in the development phase revealed that column temperature and flow rate did not significantly affect the ATPs. However, the ethanol concentration in the mobile phase and formic acid concentration in the aqueous phase demonstrated significant influences on peak properties. As a result, these factors were selected for further investigation: ethanol composition (Factor 1) and formic acid concentration in the aqueous phase (Factor 2).

#### Study of CMPs on CAAs using DoE

To determine the interaction between two or more CMPs on CAAs, we employed Response Surface Methodology (RSM). Among various RSM techniques, rotatory Central Composite Design (rCCD) was chosen for its advantages, particularly its ability to create a design space from central points to extended axials (+ α and − α). The application of two factors in the rCCD generated thirteen experimental runs, which were subsequently applied to the UPLC system. The results obtained from these UPLC runs were then analyzed using software to investigate the impact of factors on the responses.

The selection of responses (CAAs) was based on the ATP. While other system suitability parameters exhibited higher values than the prescribed limits, the Rs between peaks 1 and 2 were chosen to establish a design space and obtain results with good retention factors and proper Rs between the first two peaks. The second factor was selected based on GAC principles, as an increase in the Rt of the third peak would increase solvent consumption. To address all these considerations, the responses were carefully selected. The resulting interactions between the factors and their corresponding responses are detailed in Table 1.

**Table 1:** Various levels of interaction between robust factors and their corresponding responses

Run	Factors		Responses	
	A: Ethanol %	B: % of Formic Acid	Rs between Peak 1 and Peak 2	Rt of Peak 3
1	57.07	0.14	4.23	2.587
2*	50.00	0.10	4.82	3.531
3*	50.00	0.10	4.74	3.548
4	50.00	0.05	4.12	5.887
5*	50.00	0.10	4.68	3.556
6	42.93	0.14	5.63	3.758
7*	50.00	0.10	4.54	3.519
8	40.00	0.10	4.72	4.905
9	42.93	0.06	3.65	6.145
10	60.00	0.10	4.94	2.772
11	50.00	0.15	4.83	3.128
12	57.07	0.06	4.76	4.23
13*	50.00	0.10	4.62	3.528

^*^Center axials of the designs

##### Impact of CMPs on resolution (Rs)

**Fig. 2:**
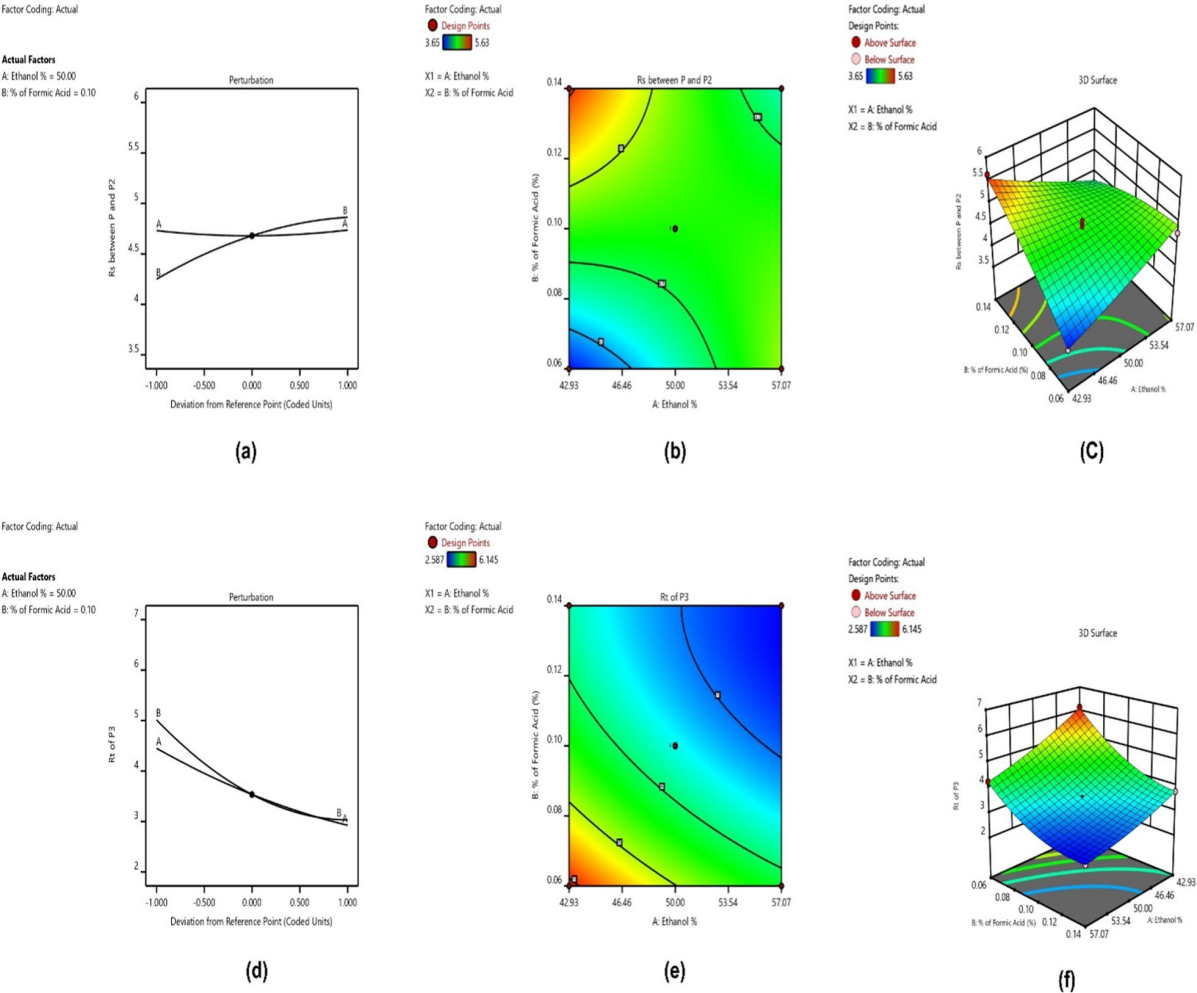
**a**, **d** Perturbation plot for factors A and B how their influence responses, **b**, **e** 2D plot for factors A and B on how their influence responses, **c**, **f** 3D plot for factors A and B how their influence of responses

The initial interpretation of CMPs' impact on CAAs was based on statistical and graphical models. In the statistical analysis, key parameters considered included p-values, F-values, lack of fit, and R^2^. The p-values for the present model were less than 0.05, indicating statistically significant interactions. The F-value of 26.41 and lack of fit p-value of 0.2057 demonstrated that the factor is important and the model fits the data well. A high R^2^ of 0.9497 indicated that the model explains most of the variation in the response. Graphical data presented in Perturbation (Fig. 2a, d), 2D (Fig. 2b, e), and 3D contour plots (Fig. 2c, f) illustrated the interaction of CMPs on Rs. Ethanol showed a nominal effect on Rs while increasing formic acid concentration resulted in increased Rs between the two drugs.

##### Impact of CMPs on RFB Rt

For the second model, p-values were also less than 0.05, indicating statistical significance. The F-value of 5470.37 and lack of fit p-value of 0.1074 demonstrated the factor's importance and good model fit. A high R^2^ of 0.9997 indicated that the model explains almost all variation in the response. Graphical data in Perturbation (Fig. 2a, d), 2D (Fig. 2b, e), and 3D contour plots (Fig. 2c, f) illustrated the interaction of CMPs on Rt of RFB. Both ethanol and formic acid concentrations significantly decreased the Rt of RFB.

The Final Equation in Terms of Coded Factors serves as a powerful tool in modeling and optimizing responses in DoE studies, providing insights into factor-response relationships. The coded equations for the two interactions were as follows.

$$\text{Rs between P and P}2\hspace{0.17em}=\hspace{0.17em}\text{Rs between P and P}2\hspace{0.17em}=\hspace{0.17em}- 7.05938\hspace{0.17em}+\hspace{0.17em}0.143883\text{ Ethanol \%}\hspace{0.17em}+\hspace{0.17em}153.98743\text{\% of Formic Acid }- 2.51017\text{ Ethanol \%}\hspace{0.17em}\times \hspace{0.17em}\text{\% of Formic Acid}\hspace{0.17em}+\hspace{0.17em}0.001075\text{ sq}.\text{ of Ethanol \% }- 99.01310\text{\% sq}.\text{ of Formic Acid}$$

$$\text{Rt of P}3\hspace{0.17em}=\hspace{0.17em}26.993-0.48788\text{ Ethanol \% }- 143.223\text{\% of Formic Acid }0.744\text{ Ethanol \%}\hspace{0.17em}\times \hspace{0.17em}\text{\% of Formic Acid }0.003056\text{ sq}.\text{ of Ethanol \% }389.891\text{\% sq}.\text{ of Formic Acid}$$

#### Derringer’s desirability

Derringer’s desirability function, a robust optimization method, evaluates responses on a scale from 0 (low effectiveness) to 1 (ideal performance). It transforms multiple responses into a single measure of desirability, facilitating optimization for both maximizing and minimizing response variables.

In this study, desirability was obtained by selecting factors and responses with the ATP in consideration. Rs between AMX and OMP was maximized, while other factors were maintained within specific limits to obtain more runs within desirability limits. This approach optimized analytical processes while minimizing environmental impact, demonstrating a commitment to sustainable practices. The responses adhered to system suitability criteria, with the first two responses falling within the acceptable range, suggesting an effective single solution. Practical application of predicted outcomes revealed deviations between practical and theoretical results within 10%, demonstrating good agreement.

#### Overlay graph and design space

**Fig. 3:**
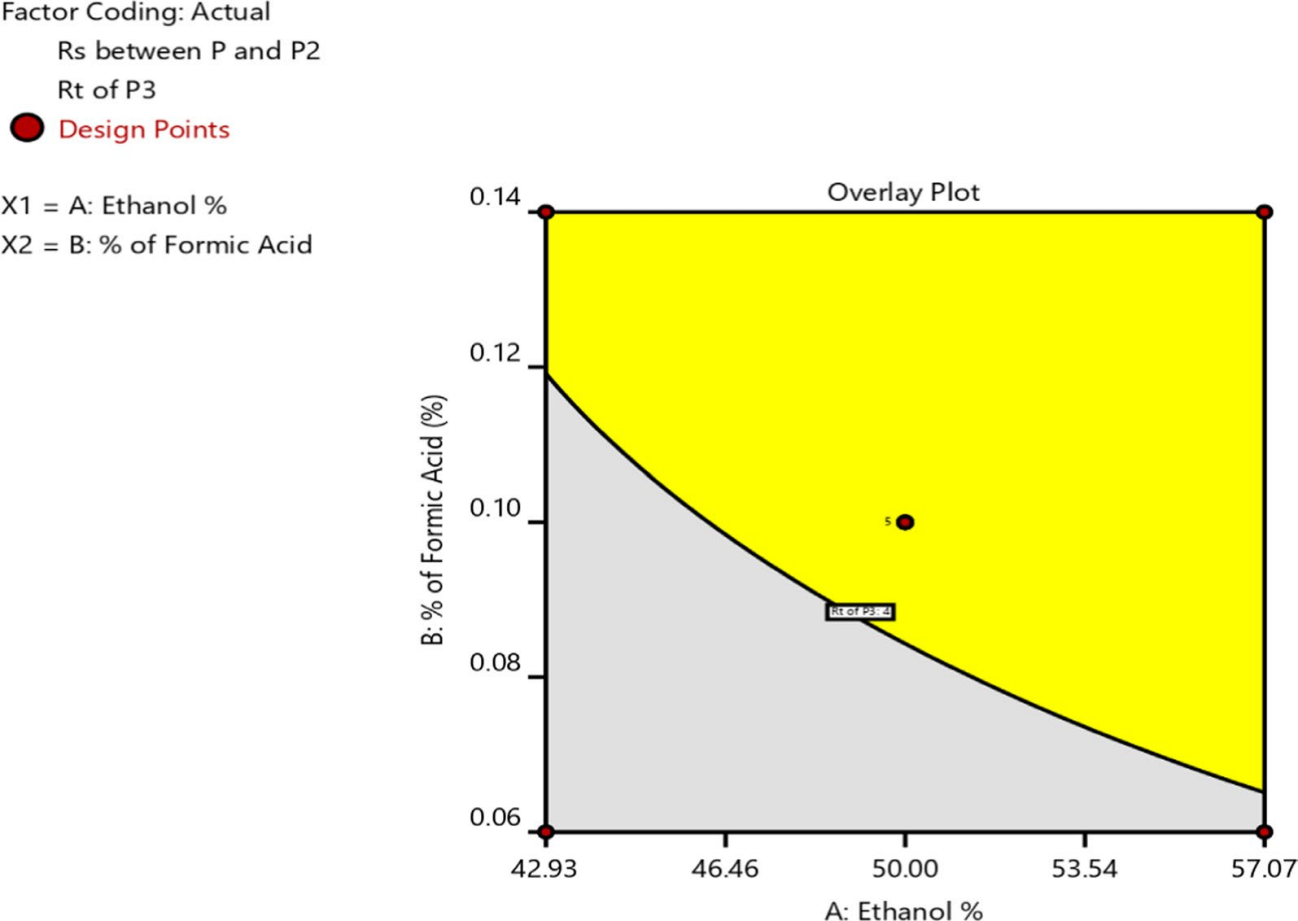
The overlay plot for the optimum approach indicates that the yellow color represents a favorable factor environment, while the grey color represents an unfavorable influence configuration

The overlay graphical tool, particularly useful in RSM, optimizes processes and elucidates the impact of variables on multiple outputs. Figure 3 shows the overlay graph, where the “yellow zone” denotes the design space, indicating areas where variations are not expected to substantially impact the proposed method’s quality. This design space serves as a guideline to identify the acceptable range of CMPs that can be adjusted without compromising desired quality attributes. By considering this design space, the method can be consistently optimized and controlled to achieve desired quality outcomes. A single method was selected from the design space and validated according to guidelines.

### Validation of the proposed method

Method validation is a documented process that offers strong assurance that a specific analytical method is appropriate for its intended use. The approved procedure was validated in accordance with the guidelines set forth in ICH Q2 (R1) of Technical Requirements for Pharmaceuticals for Human Use.

#### System suitability test

The system suitability test involved injecting six replicates of standard solutions containing 20 (OMP), 500 (AMX), and 25 μg/ml of RFB. Key systems of measurement such as standard deviation, and % RSD were assessed for each injection. Table 2 summarizes the results, indicating that the % RSD values were consistently below 2% across all replicates, confirming the method's suitability for repeatability even after multiple sample analyses.

**Table 2:** System suitability parameters for OMP, AMX, and RFB

Parameters	AMX			OMP			RIF		
	Average	SD	% RSD	Average	SD	% RSD	Average	SD	% RSD
Rt	1.3694	0.02	1.41	2.6596	0.02	0.58	3.73	0.02	0.43
Peak area	2,965,352	20,022.16	0.68	113,389.8	831.88	0.73	144,364	1699.19	1.18
K’ (AMX) Rs (OMP & RIF)	2.91	0.06	1.93	5.26	0.10	1.92	4.90	0.07	1.44
Tailing factor	1.3475	0.02	1.86	1.548	0.03	1.91	1.17	0.02	1.65
Theoretical plates	11,768.8	110.26	0.94	6048.6	15.03	0.25	7218.60	8.79	0.12

#### Linearity, limits of detection (LOD) and quantification (LOQ)

An analytical method is considered linear if it produces results that, within a specified range, are directly proportional to the analyte concentration or can be mathematically transformed to be proportional. To assess linearity, varying quantities of standard drug solutions were injected in six replicates to achieve concentration ranges of 5–30 µg/ml for OMP, 125–750 µg/ml for AMX, and 6.25–37.50 µg/ml for RFB. Standard linear regression analysis was employed to evaluate the linearity by comparing the recorded peak areas with the corresponding drug concentrations. The slope, correlation coefficient (r^2^), and intercept (along with their respective confidence intervals), were calculated and evaluated. ICH guidelines outline several methods for determining the LOD and LOQ based on the slope, using the signal-to-noise ratio and the standard deviation of the response. Following ICH recommendations, the LOD and LOQ for this study were calculated and are detailed in Table 3.

**Table 3:** Linearity results for the validation of the proposed method

Parameters	AMX	OMP	RFB
Concentration range (μg/ml)	125 to 750	5 to 30	6.25 to 37.50
r^2^	0.9995	0.9993	0.9997
Y =	5831.6x + 25,304	5594.6x + 2999.5	5819x + 979.47
LOD (μg/ml)	0.616	0.026	0.037
LOQ (μg/ml)	1.866	0.078	0.113
Accuracy (Mean ± % RSD)	99.29 ± 0.85	99.47 ± 1.85	99.91 ± 1.58
Precision			
Intraday (% RSD)	1.111	0.78	0.92
Interday (% RSD)	0.845	0.94	0.81

#### Accuracy

It is often possible to enhance the accuracy of the UPLC method, which also addresses variations in the detector’s response. The accuracy was evaluated using the conventional addition approach. Additional quantities corresponding to 50 (10 µg/ml for OMP, 250 µg/ml for AMX, and 12.5 µg/ml for RFB), 100 (20 µg/ml for OMP, 500 µg/ml for AMX, and 25 µg/ml for RFB), and 150% (30 µg/ml for OMP, 750 µg/ml for AMX, and 37.5 µg/ml for RFB) levels of the typical concentrations of OMP, AMX, and RFB were added to pre-quantified sample solutions. The developed method was then applied to analyze these mixtures. The experiment was conducted in six runs. For each concentration, the percentage recovery, %RSD, and percentage were calculated to assess the method's accuracy and found to be within the limits and the results are depicted in Table 3.

#### Precision

The precision of the developed method was evaluated through intra-day and inter-day precision studies, ensuring consistent and reproducible results over short and extended periods. Intra-day precision was assessed by analyzing three replicates of three different concentrations on the same day. The %RSD of the observed peak areas was calculated and reported as a measure of precision. For inter-day precision, the same concentrations of the drugs were analyzed in triplicate over three separate days, and the %RSD was calculated and depicted in Table 3 shows the developed methods approach ensured a comprehensive assessment of the method’s precision over time.

#### Selectivity

The method's selectivity was validated by analyzing and comparing chromatograms from standard solutions, sample solutions, and the relevant placebo samples. This comparison ensured that the method accurately identified and quantified the analytes in the presence of other components.

## Forced degradation results

**Fig. 4:**
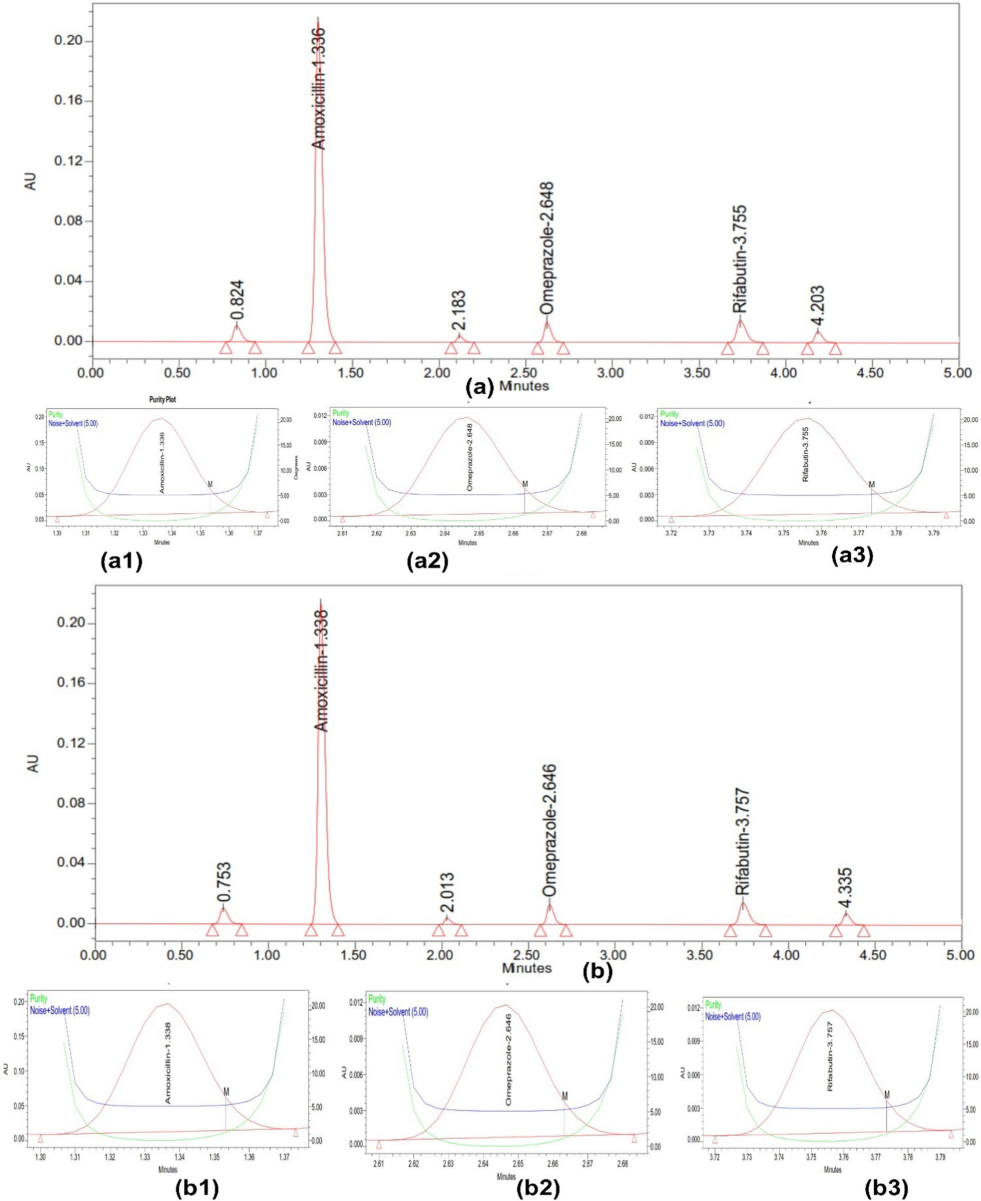
Stress study of three drugs using (**a**) Acid and (**b**) Base, along with their peak purity for (**a1**,** b1**) AMX (**a2**,** b2**) OMP (**a3**,** b3**) RFB

**Fig. 5:**
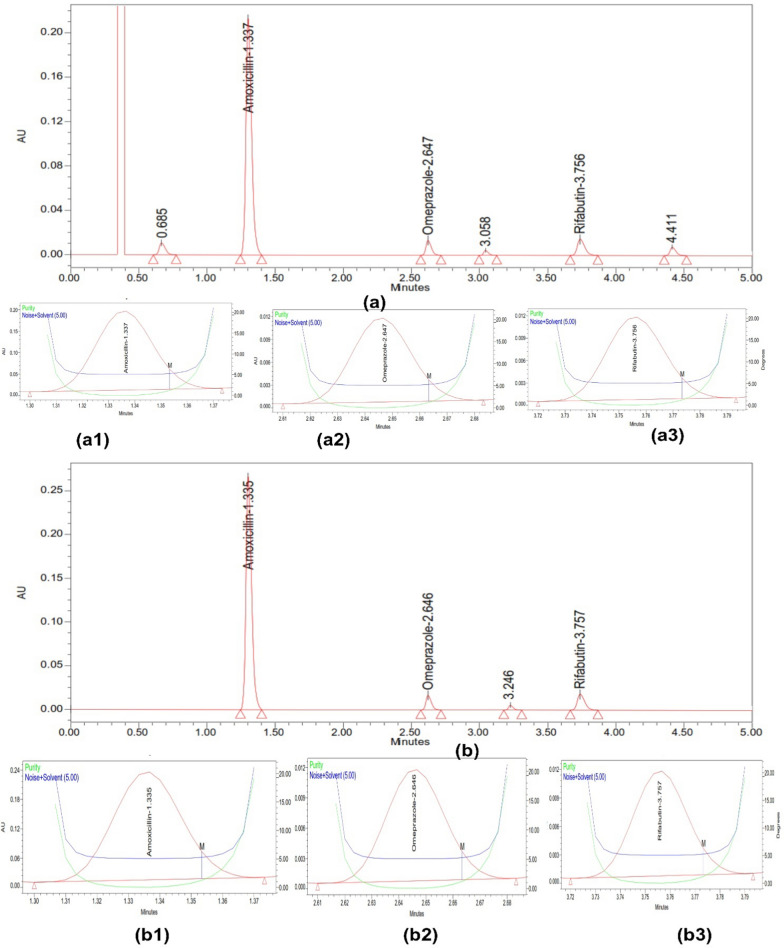
Stress study of three drugs using (**a**) Oxidation using H_2_O_2_ and (**b**) Reduction Using NaHSO_3_, along with their peak purity for (**a1**,** b1**) AMX (**a2**,** b2**) OMP (**a3**,** b3**) RFB

**Fig. 6:**
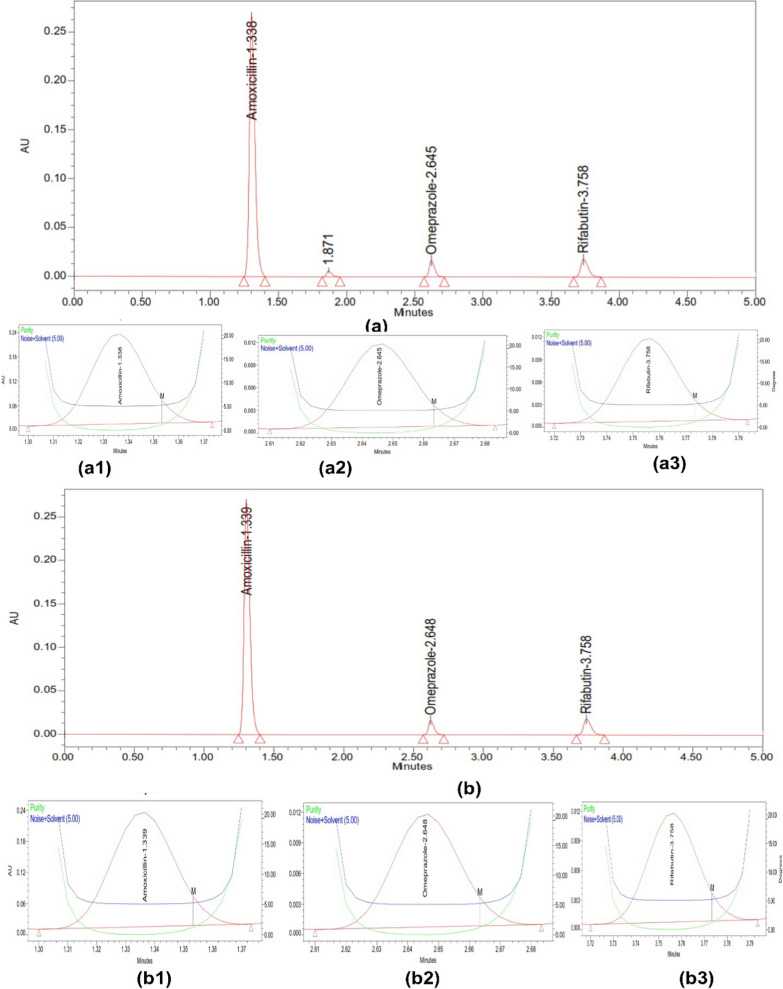
Stress study of three drugs using (**a**) Heat at 105 °C and (**b**) Photo at 72 lx, along with their peak purity for (a1, b1) AMX (a2, b2) OMP (a3, b3) RFB

The results of the forced degradation studies for RFB, AMX, and OMP under various stress conditions revealed that these drugs exhibited different degradation patterns. In acidic conditions, OMP showed significant degradation of approximately 20% over 3 h at room temperature, highlighting the importance of applying a delayed-release coating for this dosage form. In contrast, AMX and RFB experienced minimal degradation under the same acidic conditions. All three drugs demonstrated nominal degradation in basic conditions over 3 h. However, the drugs showed more pronounced degradation under oxidative stress compared to reductive stress. Light and heat did not cause significant degradation, indicating that the drugs are stable under these conditions. The overall results of the forced degradation studies are presented in Table 4, with chromatograms displaying the drug peaks and degradation peaks shown in Fig. 4 (acid and base degradation), Fig. 5 (redox degradation), and Fig. 6 (thermal and photodegradation).

**Table 4:** Degradation data of OMP, AMX and RFB

Description	Drug peak area			% Recovery (assay)			% Total degradation			Purity angle			Purity threshold		
	AMX	OMP	RFB	AMX	OMP	RFB	AMX	OMP	RFB	AMX	OMP	RFB	AMX	OMP	RFB
Standard	2,905,352	113,390	140,364	100	100	100	0	0	0	1.341	5.975	2.078	7.857	13.452	9.564
Acid (3 h)	2,701,234	91,675	127,579	92.97	80.85	90.89	7.03	19.15	9.11	1.363	5.947	2.064	7.832	13.462	9.546
Alkali (3 h)	2,622,501	101,763	126,845	90.26	89.75	90.37	9.74	10.25	9.63	1.375	5.969	2.032	7.818	13.447	9.589
Peroxide (3 h)	2,544,512	98,462	124,247	87.58	86.83	88.52	12.42	13.17	11.48	1.328	5.952	2.057	7.846	13.416	9.572
Reduction (3 h)	2,654,218	109,567	129,856	91.36	96.63	92.51	8.64	3.37	7.49	1.354	5.955	2.049	7.874	13.438	9.583
Thermal (3 h)	2,654,218	110,095	137,895	91.36	97.09	98.24	8.64	2.91	1.76	1.369	5.972	2.026	7.862	13.459	9.588
Photolytic (72 lx)	2,885,412	111,462	139,967	99.31	98.30	99.72	0.69	1.70	0.28	1.335	5.948	2.084	7.829	13.441	9.516

## Greenness assessment for the developed

It is essential to conduct a greenness assessment before claiming that a methodology is environmentally friendly. The proposed technique was evaluated using different green assessment approaches to confirm the scientific validity and sustainability of the developed method.

### Solvent selection by using Hansen space for the proposed method

The Green Solvent Selection Tool (http://green-solvent-tool.herokuapp.com/) is a web-based resource that assists researchers in identifying eco-friendly solvents. By utilizing the Hansen Solubility Parameter (HSP) framework, it assesses the interaction between solvents and solutes based on factors like hydrogen bonding, polar forces, and dispersion forces [19, 20]

The tool allows researchers to compare HSP values of different solvents and solutes to determine compatibility, which helps in selecting the most appropriate solvents for various applications. Users can input the HSP values of their solutes or target compounds, and the tool generates a list of compatible solvents. This methodology supports the selection of solvents that are less harmful to the environment and can enhance process efficiency.

**Fig. 7:**
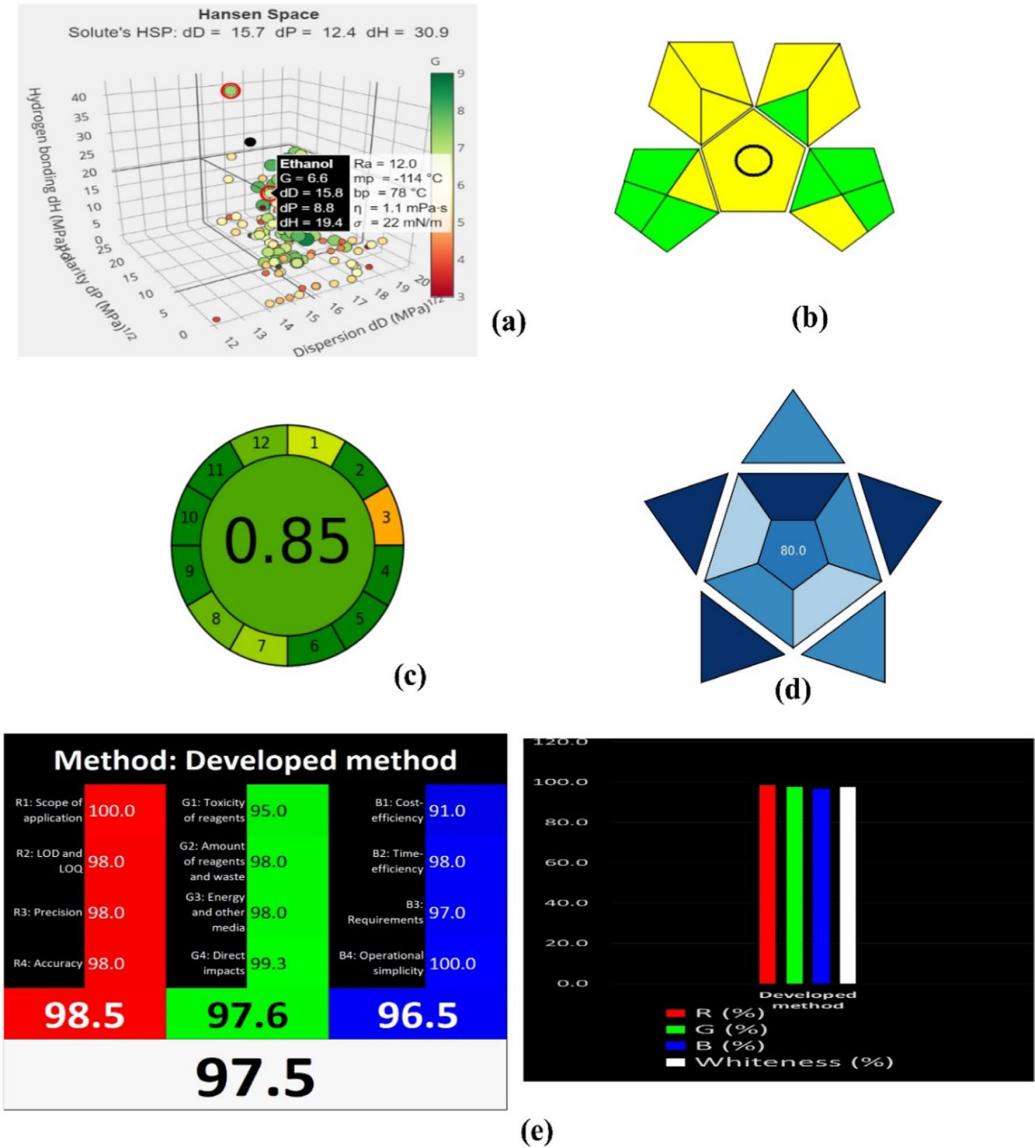
Greenness assessment for the proposed method by using (**a**) Hansen Space (**b**) GAPI (**c**) AGREE metrics (**d**) BAGI (**e**) WAC assessment

In this study, ethanol was chosen for method development using the tool, which yielded a G score of 6.6, closely aligning with the G score of 7.3 for water (Fig. 7a). The average G score for the ethanol–water combination was 6.95, indicating that the chosen solvent is environmentally friendly and approaches the maximum G score of 10. This analysis verifies the appropriateness of the selected solvent for conducting environmentally sustainable research.

### Green analytical procedure index (GAPI)

The GAPI is a qualitative tool designed to assess the environmental sustainability of analytical methods. It uses visual indicators to semi-quantitatively evaluate the ecological impact of these procedures. By incorporating eco-friendly ethanol solvents into the methodology, a green-rated method was developed, emphasizing its environmentally conscious attributes.

The GAPI assessment is represented visually through a color-coded pictogram, which indicates the method’s eco-friendliness. The GAPI evaluation framework, which includes 15 distinct stages, is thoroughly incorporated into the GAPI software interface. The method was evaluated using this software, and the pictogram predominantly displayed yellow and green areas, underscoring its environmental viability [21–25].

For instance, the total reagent volume used was under 1 ml, with the entire analysis completed in 5 min at a flow rate of 0.2 ml/min, utilizing only 1 ml of mobile phase, of which 0.53 ml is the organic phase. Furthermore, the UPLC energy consumption is less than 0.1 KWh, which is also represented by green in the pictogram. The results of the GAPI assessment are detailed in Fig. 7b.

### Analytic GREEnness (AGREE)

The AGREE metrics is an advanced tool designed for comprehensive evaluation of the environmental impact of analytical methods. It offers an in-depth assessment by visualizing results in a circular graph divided into 12 segments, each representing one of the twelve principles of green analytical chemistry. Each segment is rated on a scale from 0 to 1, with 1 indicating the highest level of environmental friendliness. The overall average score is displayed at the center of the graph, with scores closer to 1 signifying greater environmental benefit. This tool is seamlessly integrated with existing methodologies, ensuring that the results highlight the most environmentally friendly approaches.

Using the AGREE tool, the current method received a score of 0.85 (Fig. 7c). This high rating is due to the use of eco-friendly solvents and the efficient energy consumption of the UPLC system, showcasing strong compliance with green analytical chemistry principles and highlighting the method’s environmental benefits. The minor reduction in the score is mainly attributed to the analytical technique being classified as at-line analysis.[26–29].

### Blue applicability grade index (BAGI) assessment

The BAGI evaluates ten attributes, each scored from 10 to 2.5, with higher scores represented by dark blue and lower scores by white [30]. These attributes are represented in a ten-part asteroid pictogram, where the inner part corresponds to Attributes 1–5 related to either the analytical determination or sample preparation steps. Attributes 6–10, relevant to both steps, are positioned in the outer part. The overall score is depicted in the central field with a color gradient based on a scale from 25 to 100, using the ‘blues’ sequential color map. This ensures a color-blind safe and legible representation, even in grayscale. Applying the present method to the BAGI software resulted in a score of 80 (Fig. 7d). This score indicates that the method is environmentally friendly and sustainable, confirming its alignment with practices that prioritize environmental consciousness in analytical methodologies.

### RGB12 whiteness assessment tool alignment with white analytical chemistry (WAC) principles

The RGB12 Whiteness Assessment Tool evaluates scientific methods using red, green, and blue hues to align with the twelve principles of WAC [31–34]. The developed RP-UPLC-PDA method achieved exceptional ratings across all categories: red for analytical efficiency, green for eco-friendliness, and blue for economic and practical efficiency. This led to an overall whiteness score of 98.5% (Fig. 7e).

The RGB12 program offers detailed data on method performance based on the 12 WAC principles and is freely available as an Excel sheet. Standardizing the application process of this tool would enhance result reliability and credibility, as subjective scoring can introduce variations in results. The high whiteness score indicates that the RP-UPLC-PDA method is highly efficient, environmentally friendly, and economically viable, making it a robust choice for analytical applications.

## Comparison of sustainability between the reported and developed methods

The simultaneous analysis of three drugs has been previously reported in two articles; however, the method requires a complete revamp for two main reasons. Firstly, the method does not adhere to current guidelines. Secondly, the method utilized toxic solvents, posing significant environmental hazards. To ensure long-term usability, a sustainability comparison between the developed and reported methods is essential.

The sustainability comparison, depicted in Table 5, indicates the developed method's superiority over previously reported methodologies. Various assessment tools were used for comparison, and the Hansen solubility parameter showed that the solvent used in the reported method had an average G score of 6.55. In contrast, the developed method achieved a G score of 6.95, closer to that of water, highlighting the credibility of selecting an eco-friendly solvent for drug analysis.

**Table 5:** Evaluation of the environmental friendliness of the proposed methods compared to the reported methods using their respective chromatographic conditions

S.No	Developed by	Chromatographic Conditions	Hansen space*	GAPI	AGREE	BAGI
1	Rama Kandula, Raja Sundararajan et, al.,	RP-HPLC 0.1 M KH_2_PO_4_ buffer (3.5 pH): acetonitrile. The flow rate of 1.0 ml/min	**5.8 + 7.3** **Avg = 6.55**			
2	Vivek Jain, Neetesh Kumar Jain et, al.,	RP-HPLC 20 mM KH_2_PO_4_: acetonitrile (pH 4.0 with OPA) in the ratio of 20:80v/v Flow rate of 1.0 ml/min	**5.8 + 7.3** **Avg = 6.55**			
3	Proposed method	UPLC Formic acid and ethanol (57: 43 (v/v))	**6.6 + 7.3** **Avg = 6.95**			

*Hansen space = G Score of Solvent 1 + Buffer (Considered G Score of water) and their average

Instrument selection is another crucial criterion. The developed method employed UPLC, which consumes less solvent and energy and generates less waste. Pharmaceutical industries adopting this method can perform analyses in an eco-friendly manner while processing more samples in less time. The advancement of the methodology is further evidenced by other assessment tools, such as GAPI, AGREE, and BAGI, showcasing the method's sustainability compared to the reported ones. The overall assessment results are presented in Table 5, demonstrating the comparative sustainability of both the reported and developed methods.

## Attaining Sustainable Development Goals through eco-friendly chromatography

The approach employed in this study directly supports several Sustainable Development Goals (SDGs) by integrating sustainability principles into the analytical method development process. Through careful optimization of chromatographic conditions and solvent usage, our findings make important contributions to sustainability in both laboratory practices and broader environmental contexts:

- *Good Health and Well-being (Goal 3)* The chromatography method developed in this study significantly reduced the use of harmful organic solvents by employing a lower volume of eco-friendly solvents. This minimizes chemical exposure for laboratory personnel and reduces the environmental impact of hazardous waste disposal, contributing to overall well-being and safety.
- *Quality Education (Goal 4)* The green analytical techniques demonstrated in this study can serve as a practical example in educational settings, enhancing the teaching of sustainable practices in analytical chemistry. These techniques can be incorporated into academic and training curricula, empowering future scientists to adopt eco-friendly practices that align with global sustainability goals.
- *Gender Equality (Goal 5)* This research involved a diverse team of scientists from different genders, promoting an inclusive research environment. The study serves as an example of how diverse collaboration can be achieved in scientific research, contributing to gender equality in scientific fields.
- *Affordable and Clean Energy (Goal 7)* In the experimental work, the use of optimized, low-energy UPLC methods contributed to reducing the energy required for analysis. The potential for future integration of clean energy sources, such as solar energy, into laboratory systems further enhances the sustainability of the methodology
- *Responsible Consumption and Production (Goal 12)* Our method’s reduced solvent use exemplifies responsible resource management, minimizing chemical waste and reducing the environmental burden on ecosystems. This approach supports the efficient use of resources in scientific research, directly contributing to more sustainable laboratory practices.
- *Climate Action (Goal 13)* By optimizing our method to lower energy consumption, we have reduced the carbon footprint of laboratory operations. This aligns with global efforts to combat climate change by mitigating greenhouse gas emissions, particularly in energy-intensive scientific processes.
- *Goals 14 (Life Below Water) and 15 (Life on Land)* By using eco-friendly solvents in the mobile phase, the study reduces solvent waste and limits the environmental impact on aquatic and terrestrial ecosystems. This supports the preservation of biodiversity and contributes to minimizing pollution that could harm water and land environments.

## Conclusion

The proposed method was meticulously designed, leveraging the principles of AQbD and confirmed conferring to ICH guidelines to ensure accuracy, precision, linearity, and specificity. The method exhibited exceptional performance in several key areas. Linearity was established for OMP, AMX, and RFB over a range of concentrations, with strong r^2^ indicating proportional responses to analyte concentrations. Precision studies, both intra- and inter-day, demonstrated low %RSD values, confirming the method’s reproducibility. Accuracy was validated through recovery studies using the standard addition method, yielding recoveries within the acceptable range, further attesting to the method’s reliability. Forced degradation studies under various stress conditions (acidic, alkaline, Redox, and photolytic) indicated that OMP, AMX, and RFB were stable, with degradation products well-separated from the analyte peaks. This underscores the method’s stability-indicating capability. Environmental sustainability was a crucial consideration in the method development. The use of ethanol as the mobile phase, selected through the Green Solvent Selection Tool, and a low flow rate of 0.2 ml/min ensured minimal solvent consumption. The method's greenness was evaluated using the GAPI, AGREE, and BAGI tools. The GAPI assessment depicted a predominantly green and yellow pictogram, indicating high environmental viability. The AGREE tool yielded a score of 0.85, reflecting strong alignment with GAC principles. The BAGI score of 80 further confirmed the method's eco-friendly attributes. Additionally, the RGB12 Whiteness Assessment Tool provided a whiteness score of 98.5%, highlighting the method's high analytical efficiency, environmental friendliness, and economic viability. The developed RP-UPLC-PDA method is not only scientifically rigorous and reliable for the simultaneous quantification of OMP, AMX, and RFB but also aligns well with sustainability goals. Its implementation can significantly enhance the efficiency and environmental sustainability of pharmaceutical analysis, setting a benchmark for future analytical method development.

## Data Availability

Data is provided within the manuscript.

## Abbreviations

OMP: Omeprazole
AMX: Amoxicillin
RFB: Rifabutin
AQbD: Analytical quality by design
GAC: Green analytical chemistry
WHO: World Health Organization
PPIs: Proton pump inhibitors
KH₂PO₄: Potassium dihydrogen orthophosphate
PDA: Photo Diode Array
Rt: Retention time
Rs: Resolution
PVDF: Polyvinylidene fluoride
CAAs: Critical Analytical Attributes
CMPs: Critical Method Parameters
DoE: Design of experiments
ATP: Analytical target profile
IFBD: Ishikawa fishbone diagram
RSM: Response Surface Methodology
LOD: Limits of detection
LOQ: Limit of quantification
HSP: Hansen Solubility Parameter
GAPI: Green analytical procedure index
AGREE: Analytic GREEnness
BAGI: Blue applicability grade index
WAC: White analytical chemistry
SDGs: Sustainable Development Goals
